# Efficacy and Acceptability of a Mobile App for Monitoring the Clinical Status of Patients With Chronic Obstructive Pulmonary Disease Receiving Home Oxygen Therapy: Randomized Controlled Trial

**DOI:** 10.2196/65888

**Published:** 2025-01-06

**Authors:** Anisbed Naranjo-Rojas, Luis Ángel Perula-de Torres, Freiser Eccehomo Cruz-Mosquera, Guillermo Molina-Recio

**Affiliations:** 1 Health and Education Research Group (GINEYSA) Faculty of Health Universidad Santiago de Cali Santiago de Cali Colombia; 2 Biomedicine Doctoral Program University of Córdoba Córdoba Spain; 3 Research Network on Chronicity Primary Care and Health Promotion (RICAPPS) Cooperative Research Networks Oriented to Health Results (RICORS) Carlos III Health Institute Madrid Spain; 4 Integral Health Research Group (GISI) Faculty of Health Universidad Santiago de Cali Santiago de Cali Colombia; 5 Nursing Pharmacology and Physiotherapy Department University of Córdoba Lifestyles Innovation and Health (GA-16) Maimonides Biomedical Research Institute of Córdoba (IMIBIC) Spain University of Córdoba Córdoba Spain

**Keywords:** m-Health, chronic obstructive pulmonary disease, quality of life, mobile health applications, home oxygen therapy, mobile phone

## Abstract

**Background:**

Chronic obstructive pulmonary disease (COPD) primarily originates from exposure to tobacco smoke, although factors, such as air pollution and exposure to chemicals, also play a role. One of the primary treatments for COPD is oxygen therapy, which helps manage dyspnea and improve survival rates. Mobile health (mHealth) technologies have demonstrated significant potential in monitoring patients with chronic diseases, offering new avenues for enhancing patient care and disease management.

**Objective:**

The purpose of this study was to evaluate the efficacy and acceptability of a mobile app designed for the clinical monitoring of patients with COPD and home oxygen (HO) therapy, compared with conventional monitoring in real-world community settings.

**Methods:**

A parallel-group, nonblinded, multicenter randomized controlled trial was conducted with 45 participants; the intervention group (IG), which used the mobile app in addition to conventional monitoring (n=23) and the control group, which received only conventional monitoring (n=22), administered by therapists over a duration of 3 months. The primary outcomes included the chronic obstructive pulmonary disease assessment test (CAT) score, the level of dyspnea measured by the Borg scale, and oxygen saturation percentage, assessed at both the beginning and end of the trial. Secondary outcomes included the frequency of app use, the number of hospitalizations, and survival rates. In addition, a satisfaction survey and an interview were conducted with the IG.

**Results:**

The median use of the mobile app was 21 (IQR 16-28) days. At the end of the follow-up, the Borg dyspnea scale was significantly lower in patients who used the mobile app for HO therapy monitoring (mean 0.6, SD 0.8 vs mean 4.1, SD 1.4; *P*=.001). Regarding the impact of COPD on quality of life, as measured by the CAT, no differences were found in the scores between baseline and end-of-follow-up within the control group. However, a significant decrease was observed in the IG (baseline median CAT 27, IQR 23-31 vs final median CAT 22, IQR 14-28; *P*<.001). In addition, the CAT score was significantly higher in patients receiving conventional monitoring compared with those monitored with the mobile app (median 30, IQR 23-32 vs median 22, IQR 14-28; *P*=.02).

**Conclusions:**

The use of the mobile app, AppO2 (SINCO), designed for the clinical monitoring of patients with COPD and HO therapy, is associated with improved quality of life. In addition, the app is highly accepted by users, promotes self-care, and fosters patient confidence in managing their own condition.

**Trial Registration:**

ClinicalTrials NCT04820790; https://clinicaltrials.gov/study/NCT04820790

**International Registered Report Identifier (IRRID):**

RR2-https://doi.org/10.1186/s12875-021-01450-8

## Introduction

The main cause of chronic obstructive pulmonary disease (COPD) is exposure to tobacco smoke; however, there are also other associated factors such as air pollution, exposure to chemicals, and a history of respiratory diseases [1]. The World Health Organization considers it the third cause of death worldwide, making it a public health problem [2]. It is estimated that approximately 8% of the Colombian population—equivalent to about 3.6 million people—suffers from COPD [3,4]. Oxygen therapy is one of the main treatments for this disease [5], aiding in the control of symptoms such as dyspnea and improving survival [5]. Mesquita et al [6] found patients with COPD who did not adhere to oxygen therapy experienced a long-term decrease in quality of life compared to those who followed their prescribed treatment [7]. Similarly, several authors [6,8,9] have identified factors such as age, communication barriers between doctor and patient, clinical follow-up, and disease knowledge as significant challenges to adhering to oxygen therapy [6]. Low adherence can lead to exacerbations [10], which deteriorate lung function, negatively affect patients’ quality of life, and increase mortality [11].

Furthermore, these exacerbations contribute to substantial health care costs. In Colombia, each exacerbation costs an average of US $98 [12], with costs rising to nearly US $700 if hospitalization is required [12]. In addition, this high cost can be attributed to the fact that once a patient is hospitalized, there is a high risk of rehospitalization or death. For example, Niewoehner [13] found that of 1016 patients hospitalized for COPD, some were rehospitalized and 33% (n=335) died within 6 months of discharge.

Conversely, mobile health (mHealth) technologies hold significant promise for monitoring patients with chronic diseases [14]. These apps offer benefits such as remote monitoring, direct communication with health care professionals, recording of clinical signs, and the development of self-care skills [14]. A Cochrane review [15] suggested that self-monitoring of symptoms through mobile apps positively impacts the development of self-care and self-management skills in patients with chronic diseases [9]. Furthermore, studies, such as the one conducted by Knox et al [16], have focused on designing mobile apps for patients with COPD, concluding that reporting clinical information through these apps can aid in disease management [16].

Some authors emphasize the importance of designing these mHealth apps with a user-centered approach [17,18]. Understanding the perceptions and needs of the end user as well as adapting to their sociodemographic characteristics and level of technological literacy are indispensable strategies for developing mHealth apps that increase adherence to use and, consequently, to treatment and self-care [17-19]. Therefore, evaluating the usability [20] of mHealth apps before their implementation is crucial. Conducting usability tests to assess user performance on specific tasks can identify areas for design improvements and enhance user satisfaction [4,21].

Based on the above, the authors of this study designed and developed a mobile app called AppO2 (SINCO) before conducting the research. This app was created using a user-centered design methodology and underwent usability tests [4,19]. AppO2 facilitates the monitoring of the clinical status of patients with COPD receiving home oxygen (HO) therapy, with 2 user profiles—patients (or their caregivers) and health care professionals [4,19]. The functions of the patient profile are centered on self-care skill development and quality of life improvement. These functions include tutorials on measuring and recording vital signs, accessing information related to the prescription of HO treatment, and communicating and interacting with health care professionals [4,19]. Conversely, the functions of the professional profile are designed to monitor patients’ clinical status on a daily basis, record and review clinical changes to inform decision-making, and control the dosage of HO therapy [4,19].

Finally, the functionalities of this mobile app were designed to promote self-care in patients with COPD receiving HO therapy. It also sought to address the primary challenges these patients face in improving adherence to HO treatment, preventing or detecting exacerbations early, and enhancing their quality of life [4,19].

In this context, this study aimed at evaluating the efficacy and acceptability of AppO2 in real community settings compared with conventional home care monitoring for patients with COPD-prescribed HO.

## Methods

### Study Design and Participants

A 3-month, open-label, 2-arm, parallel-arm, multicenter, randomized controlled, nonblinded, clinical trial was conducted to determine the efficacy of a mobile app in monitoring the clinical status of patients with COPD receiving HO therapy. The control group (CG) comprised patients receiving HO and monitored using conventional methods, such as weekly home visits by a health care professional. The intervention group (IG) comprised patients who were also monitored through weekly home visits in addition to using the AppO2 mobile app. The clinical trial was conducted in Cali, Colombia, with the participation of 3 home care institutions—TodoMed, Amanecer Médico, and Cuidarte en Casa. The clinical trial protocol was registered in ClinicalTrials.gov (ID NCT04820790) [4].

Patients were recruited from May to December 2023 based on the following inclusion criteria: (1) being ≥18 years old, (2) having a medical prescription for HO for more than 1 year, (3) having a caregiver, (4) owning a smartphone, and (5) consenting to audio recording. The exclusion criterion was patients undergoing mechanical ventilation.

In total, 2 health care professionals were included based on the following inclusion criteria: (1) being ≥18 years old, (2) being respiratory therapists and physiotherapists affiliated with a home care company, (3) having more than 6 months of experience in home care, and (4) having a smartphone. The exclusion criterion was established as professionals with less than 6 months of experience in managing patients receiving HO.

### Sample Size

To achieve a 1:1 ratio between the IG and CG, with 80% power (β error=20%) and 95% CI (α error=5%), and estimating that the AppO2 app in the IG would result in a 3% increase self-management of dyspnea, with SD 6 [22], a minimum sample of 32 participants (16 in each group) was estimated. Finally, to minimize the effects of potential patient losses that would reduce statistical power, the sample was expanded to a final size of 45 participants (23 in the IG and 22 in the CG) [4].

### Randomization

#### Overview

Participants were equally and randomly assigned to either the IG or CG using a simple randomization method with Epidat 3.1 (Consellería de Sanidade de la Xunta de Galicia, in collaboration with the Pan American Health Organization [PAHO]). Blinding was not possible for participants or health care professionals owing to the intervention method used.

#### Sample Selection

Consecutive sampling was used to recruit patients and all participants who met the selection criteria and attended consultations at participating institutions were included in the study. Sampling was done until the predetermined sample size was reached.

### Intervention

After selecting the trial participants, the IG downloaded the AppO2 app on their mobile phones. The objectives of the investigation and the operation of the app were explained to them. In addition, it was recommended to use the app at least once a week. All recorded information was stored anonymously in a cloud-based database, identifiable by a code only known to the principal investigator. The database was accessible to researchers through a web app for review and analysis.

The AppO2 mobile app consists of 2 user profiles, one for patients and caregivers and another for professionals. There are 3 sections in the patient and caregiver profile, “My Profile,” “Medical Visit,” and “Tutorials.” In the “My Profile” section, patients can record their vital signs and the degree of respiratory difficulty, view the medical prescription for oxygen therapy including the duration of the oxygen therapy regimen, and record the number of daily inhalations if they have a prescription for medication. When recording their vital signs, patients from their user profile receive notifications informing them if vital signs are outside the predetermined ranges. These alerts suggest that they tell their health care professional to adjust treatment as necessary or recommend that they carefully follow medical indications if a slight alteration in vital signs is detected. However, these notifications are not sent to medical staff in real-time. Instead, professionals can view patients’ clinical history as they see fit through their profile [4,19]. This functionality allows them to monitor the behavior of vital signs and make the appropriate adjustments during scheduled home visits, accessible through the “Medical Visit” section. This approach allows for more flexible management and avoids an overload of unnecessary alerts, which could generate “false positives” by prioritizing intervention based on a comprehensive patient assessment. The “Medical Visit” section allowed users to view the schedule of upcoming medical visits and the history of past visits. In addition, the “Tutorials” section featured videos that provided education and instructions on taking vital signs and using oxygen systems [4,19].

**Figure 1:**
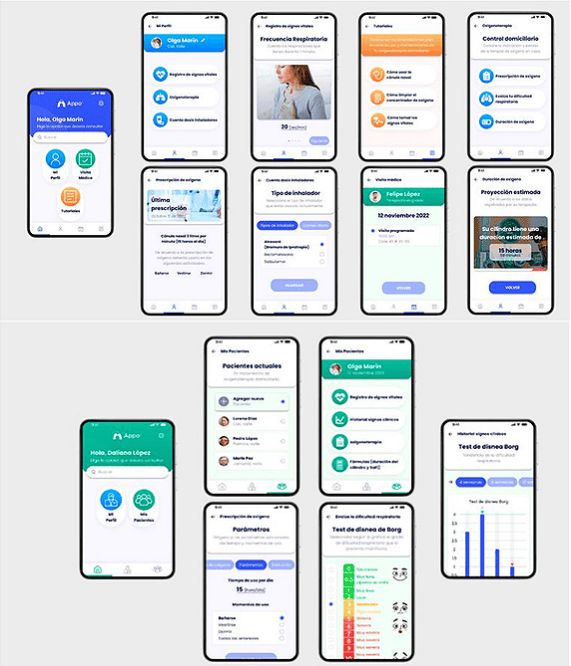
AppO2 user profiles. (A) Patient and caregiver profile. (B) Professional profile.

Similarly, the professionals’ profile has 2 sections, “My profile,” where they can register their personal data and update their account, if necessary, and “My patients,” where they can record the assessment, prescribe oxygen, and monitor the clinical status of patients. They can also view graphs that display the monthly trends of each vital sign. Through the professionals’ profile, information on HO prescription parameters, visit schedules, and data on the estimated duration of oxygen cylinders are sent to the IG [4] (Figure 1).

### Measures

Independent variables, including participants’ sociodemographic characteristics (eg, age, sex, marital status, educational level, and place of residence) and other aspects of lifestyle and treatment (such as smoking, biomass exposure, and duration of HO treatment), were obtained from the databases and record systems of the 3 health institutions to which they belonged.

During the home visits, therapists collected information was collected on variables related to vital signs, such as oxygen saturation through pulse oximetry, respiratory rate, central heart rate, number of exacerbations, number of hospitalizations, number of emergency room admissions, survival, inhaler use, and changes in oxygen prescription. Cylinder duration time was calculated considering the conversion factor, oxygen system flow, residual pressure, and cylinder pressure [23]. This calculation was performed manually in the CG, whereas the mobile app was used in the IG. In addition, therapists reminded patients and caregivers to use the app during each visit. Vital signs and the relationship between oxygen saturation and fraction of inspired oxygen [24] were recorded once a week from the date of randomization until week 12 or until the time of death from any cause, whichever occurred first.

The degree of respiratory distress was determined using the Borg Dyspnea Scale [25], a standardized, validated analog scale in Spanish, widely used since the 1970s. The scale ranges from 0 to 10 and is designed to quickly and easily assess patients’ perception of dyspnea. It includes a graph associated with each quantitative value, which helps patients identify their level of respiratory distress, with 10 indicating the highest level of perceived dyspnea [26]. Assessment using the Borg test was conducted from the beginning of the study until week 12 or until the date of death from any cause, whichever occurred first. In addition, the number of days the app was used, access to each screen, and connection time within the mobile app were tracked in the IG.

### Questionnaires

The chronic obstructive pulmonary disease assessment test (CAT) questionnaire was used to assess the impact of COPD on patients’ quality of life [27]. This questionnaire was administered to both the IG and CG at the beginning and at the end of the intervention. The acceptance of AppO2 was evaluated using the technology acceptance model (TAM) [28], which assessed the perceived usefulness and ease by participants in relation to AppO2 [28]. This questionnaire, along with an interview to gauge the perception of AppO2, was administered to health care professionals and the IG at the end of the follow-up period.

#### Chronic Obstructive Pulmonary Disease Assessment Test

This is a validated [29,30] and publicly accessible questionnaire consisting of 8 questions, each evaluated on a scale of 0-5 points. The minimum score is 0, and the maximum score is 40 [29,30], reflecting the impact COPD has on a patient’s quality of life. The scores are classified as (1) low impact (1-10 points), where most days are “good days”; (2) medium impact (11-20 points), with few “good days”; (3) high impact (21-30 points), with no “good days”; and (4) very high impact (31-40 points), where the disease’s limitation is at its maximum. A progressive increase in CAT scores indicates an increase in the impact of COPD on the patient’s quality of life [27,29].

#### AppO2 Acceptance Questionnaire and Perception Interviews

The AppO2 mobile app acceptance questionnaire was administered to patients and professionals. It was based on the TAM [30] designed by Davis [31,32], aimed at evaluating people’s perception of usefulness and ease regarding acceptance in the use of devices or software in digital environments. Although several variations have been made by different authors [33-35], it is essential to evaluate the following dimensions: (1) perception of usefulness and (2) perception of ease. Based on these dimensions, 12 items translated and validated into Spanish [36,37] were applied, with minor adaptations in their content for better understanding.

Responses were assessed using a Likert scale, ranging from “totally agree” to “totally disagree” (Multimedia Appendix 1). To determine the acceptance of the AppO2 mobile app, 1 point was assigned to the item with which participants were moderately or totally in agreement, and 0 was assigned to items with which they were moderately or totally in disagreement. Cutoff points for the level of acceptance were obtained by measuring quartiles. Low acceptance was considered if it was in quartile 1 (1-3 points), moderate if it was between quartiles 2 and 3 (4-9 points), and high if it was in quartile 4 (10-11 points). Furthermore, to complete the evaluation of participants’ perceptions regarding the AppO2 app, an interview was conducted with both the IG and health care professionals. The objective of the questions was to inquire about what they liked or disliked most about the app, the perceived benefits, and their preferences. These interviews were conducted at the end of the intervention (Multimedia Appendix 2).

### Primary and Secondary Outcomes

Primary outcomes consisted of the CAT score, the degree of dyspnea measured using the Borg test, and the percentage of oxygen saturation at the beginning and end of the clinical trial. Secondary outcomes were the frequency of use of the app, number of hospitalizations, and survival.

### Statistical Analysis

Data analysis was performed using SPSS Statistics software, version 25.0 (IBM Corp). Initially, a descriptive analysis of the study variables was conducted, characterizing the patients and generating graphs and tables of absolute and relative frequencies for qualitative variables. For quantitative variables, measures of central tendency and position (mean and median) with their respective measures of dispersion (SD and IQR) were calculated.

In the bivariate analysis, the Student *t* test or its nonparametric equivalent (Mann-Whitney *U* test) was used to compare 2 means. For categorical variables, the chi-square test was used. To analyze 3 or more mean values, repeated measures analysis of variance or the Friedman test was used. Crude odds ratios (ORs) were initially calculated to determine variables independently associated (AppO2 use and sex) with the impact of COPD on quality of life, as measured by the CAT. In addition, an analysis of covariance (ANCOVA) was used, which allowed for adjustment of differences in the final Borg dyspnea scores based on the initial baseline score. This ensured that observed changes were attributed to the intervention with the app rather than initial variations in dyspnea.

Finally, a binary logistic regression model was applied, including variables with a *P* value <.20 in the initial analysis presented or those related to the outcome by biological plausibility. This allowed for estimating the standardized β coefficients, an adjusted coefficient of determination, and the residual values. The binary logistic regression analysis dichotomized the CAT outcome into “low” (patients with a low or medium CAT score) and “high” (patients with a high or very high CAT score). The model was adjusted for possible confounding variables (time of oxygen use and years of cigarette consumption). An α error of ≤0.05 was considered statistically significant, and 95% CIs were calculated.

### Ethical Considerations

The clinical trial protocol was approved by the Ethics Committee of the Department of Health of Universidad Santiago de Cali, Colombia, Act No. 02, as well as by the 3 home care institutions in the city of Cali, Colombia. The investigation complies with international regulations, such as the Declaration of Helsinki. All participants gave their written informed consent before inclusion in the study. Informed consent was obtained, and the participants were informed of the possibility of opting out or withdrawing at the time of their choice, if applicable. The data were anonymized so that participants could not be identified, and no amount of compensation was offered or awarded.

## Results

**Figure 2:**
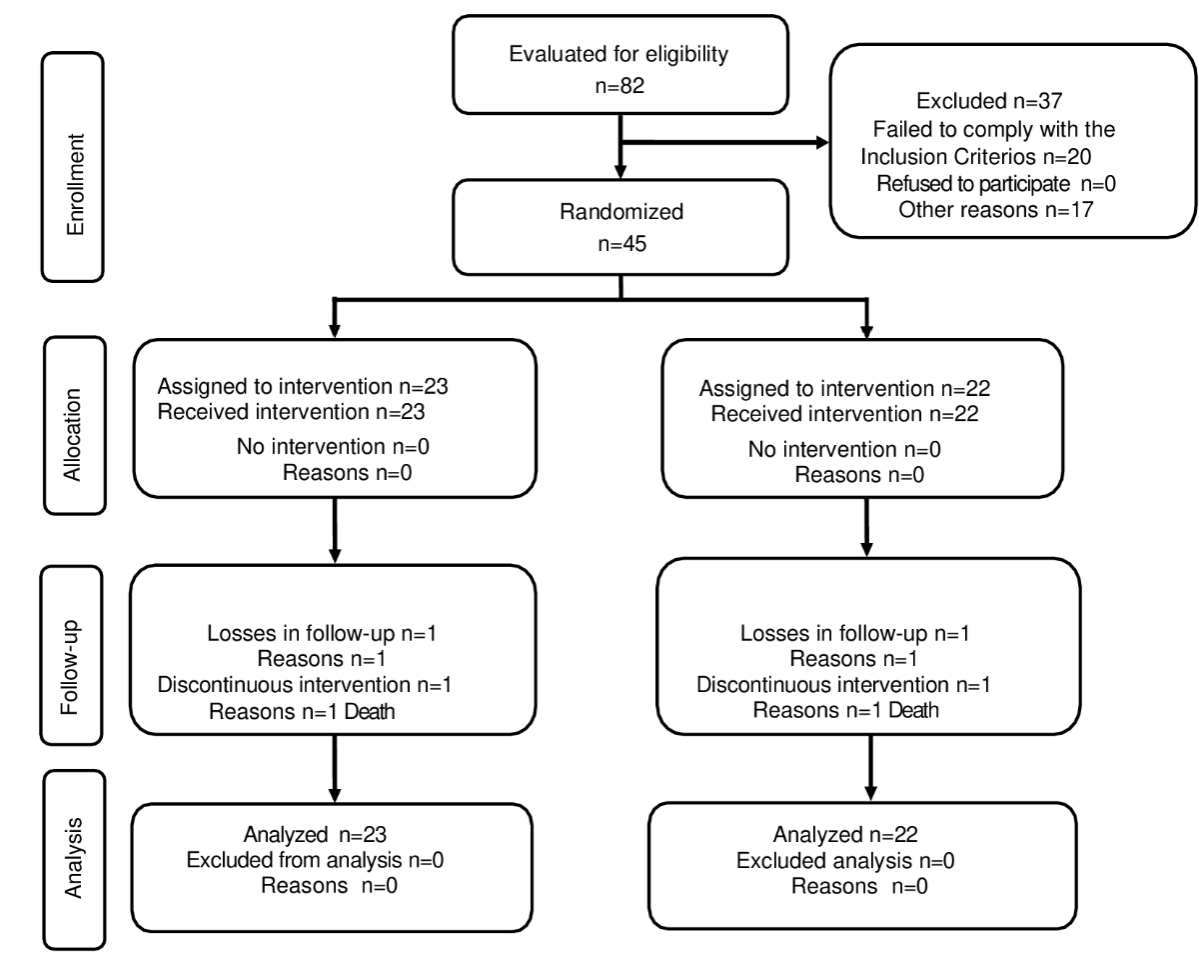
Modified CONSORT (Consolidated Standards of Reporting Trials) flow diagram for individual randomized controlled trials of nonpharmacological treatments.

Participants were equally and randomly assigned to either the IG or CG using a simple randomization method with Epidat 3.1 (Consellería de Sanidade de la Xunta de Galicia, in collaboration with the Pan American Health Organization [PAHO]). Blinding was not possible for participants or health care professionals owing to the intervention method used (Figure 2). The CONSORT Checklist is included as Multimedia Appendix 3.

A total of 45 participants were included (23 from the IG and 22 from the CG). The cohort was predominantly female (n=31, 69%) with a mean age of 75.4 (SD 15.9) years (95% CI 70.7-80.0 years). Furthermore, 73% (n=33) of the participants had been exposed to biomass at some point in their lives, and 93% (n=42) had a history of smoking, with a time of consumption that exceeded 15 years in most cases (n=30, 67%). When comparing baseline characteristics between the groups, no statistically significant differences were observed in sociodemographic variables and background except for the “time of oxygen use for more than 15 years,” which was greater in the IG (n=17, 74% vs n=9, 41%, *P*=.02; Table 1).

**Table 1:** Baseline characteristics of patients in the control and intervention groups enrolled in the study (n=45).

Variable			Intervention (n=23)		Control (n=22)		*P* value	
**Sex, n (%)**								.50^a^
	Male	8 (35)		6 (27)				
	Female	15 (65)		16 (73)				
**Education level, n (%)**								.50^a^
	Primary	18 (78)		16 (73)				
	Secondary	5 (22)		5 (23)				
	University	0 (0)		1 (4.5)				
**AppO2 usage time, n (%)**								<.001^a^
	More than 15 hours	17 (74)		9 (41)				
	Less than 15 hours	6 (26)		13 (59)				
**Activities using AppO2, n (%)**								.20^a^
	Bathing	0 (0)		0 (0)				
	Dressing	2 (9)		0 (0)				
	Sleeping	7 (30.4)		4 (18)				
	Moving around	7 (30.4)		7 (32)				
	All of the above	7 (30.4)		11 (50)				
**Biomass exposure, n (%)**								.90^a^
	Yes	17 (74)		16 (73)				
	No	6 (26)		6 (27)				
**Smoking history, n (%)**								.08^b^
	Yes	20 (87)		22 (100)				
	No	3 (13)		0 (0)				
**Years of smoking, n (%)**								.30^a^
	15 years or less	9 (39)		6 (27)				
	15 years or more	14 (61)		16 (73)				
**Institution, n (%)**								<.001^a^
	TodoMed	10 (43.5)		11 (50)				
	Amanecer médico	3 (13)		9 (41)				
	Cuidarte en casa	10 (43.5)		2 (9)				
Age (years), mean (SD)			72.1 (18.2)		78.9 (13)		.15^c^	
Baseline Borg score, mean (SD)			1.2 (0.91)		4.3 (1.4)		<.001^c^	
Baseline COPD^d^ assessment test score, mean (SD)			26.3 (8.2)		28.9 (6.9)		.28^c^	

^a^Chi-square.

^b^Continuity correction.

^c^Mann-Whitney *U* test*.*

^d^COPD: chronic obstructive pulmonary disease.

**Figure 3:**
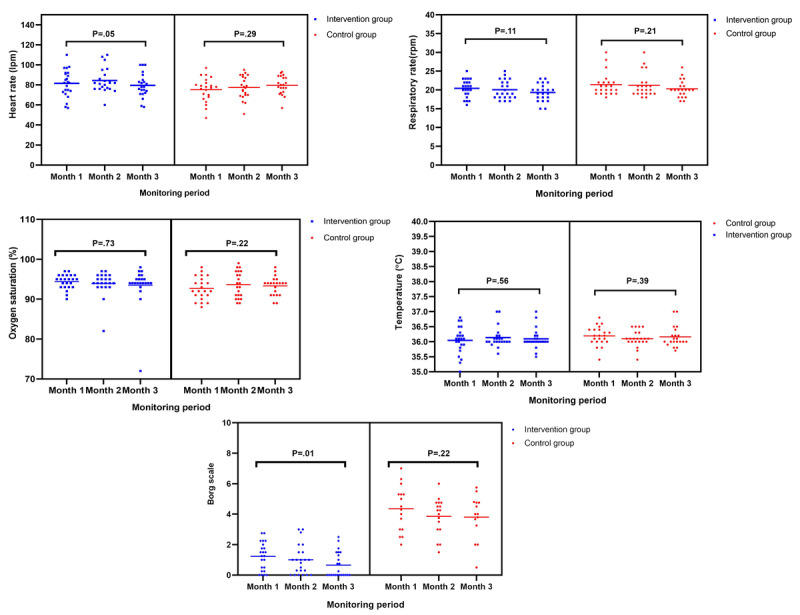
Clinical behavior during the follow-up of patients included in the study (n=45).

Regarding the behavior of vital signs during the 3-month follow-up for patients with conventional follow-up, no significant differences were observed in respiratory rate, heart rate, temperature, saturation, and Borg Dyspnea Scale scores. Likewise, in the IG, no statistically significant differences were found in vital signs, except in the Borg Dyspnea Scale score, which decreased over time (month 1: mean 1.2, SD 0.9; month 2: mean 1.0, SD 0.9; month 3: mean 0.6, SD 0.8; *P*=.01) (Figure 3).

When comparing the clinical variables at the end of follow-up between the groups, it was found that the Borg Dyspnea Scale score was significantly lower in the IG than in the CG (mean 0.6, SD 0.8 vs mean 4.1, SD 1.4; *P*=.001; Table 2).

After adjustment for baseline values, significant differences were maintained in both Borg Dyspnea Scale and CAT final scores, favoring the intervention group, with lower adjusted means (1.3 vs 2.7; *P*=.02 and 22.33 vs 26.69; *P*=.002, respectively; Table 3).

**Table 2:** Comparison of clinical variables of patients in the intervention and control groups (n=45).

Variable	Intervention (n=23), mean (SD)	Control (n=22), mean (SD)	95% CI	*P* value	Intervention (n=23), mean difference^a^ (SD)	Control (n=22), mean difference^a^ (SD)	*P* value^b^
Heart rate	79.5 (11.8)	79 (9.1)	–5.6 to 6.6	.87	–3.1 (2.19)	4 (2.80)	.10
Respiratory frequency	19 (2.3)	20 (2.3)	–2.3 to 0.3	.15	–1 (0.71)	–1 (0.71)	.99
Oxygen saturation	93 (5)	93 (2.3)	–2.2 to 2.2	.95	–1 (0.71)	–1 (0.71)	.99
Temperature	36.0 (0.3)	36.1 (0.3)	–027 to 0.07	.30	0 (0)	–0.1 (–0.07)	—^c^
Borg score	0.6 (0.8)	4.1 (1.4)	–4.17 to –2.83	.001	–0.6 (0.42)	–0.8 (0.56)	.72

^a^Mean difference: mean at the end−mean at the beginning of follow-up.

^b^Student *t* test.

^c^Not applicable.

**Table 3:** Analysis of covariance of the impact of the use of AppO2 on Borg Dyspnea Scale and chronic obstructive pulmonary disease assessment test scores at the end of follow-up, monitoring for the baseline levels of dyspnea.

		Mean (SD)	95% CI	*P* value^a^	Mean (SD)	95% CI	*P* value^b^
**Borg Dyspnea Scale^c^**				<.001			.02
	Intervention group (n=23)	0.65 (0.8)	0.29-1.01		1.3 (1.2)	0.77-1.84	
	Control group (n=14)	3.8 (1.4)	2.96-4.64		2.7 (1.6)	1.83-3.60	
**COPD^d^ Assessment Test (CAT)**				.01			.002
	Intervention group (n=23)	21.41 (9.01)	17.41-25.41		22.33 (4.27)	20.49-24.17	
	Control group (n=14)	27.67 (7.17)	24.4-30.93		26.69 (4.2)	24.80-28.50	

^a^Student *t* test.

^b^Analysis of covariance (ANCOVA).

^c^*R*^2^ adjusted for baseline Borg score: 0.766.

^d^COPD: chronic obstructive pulmonary disease.

**Figure 4:**
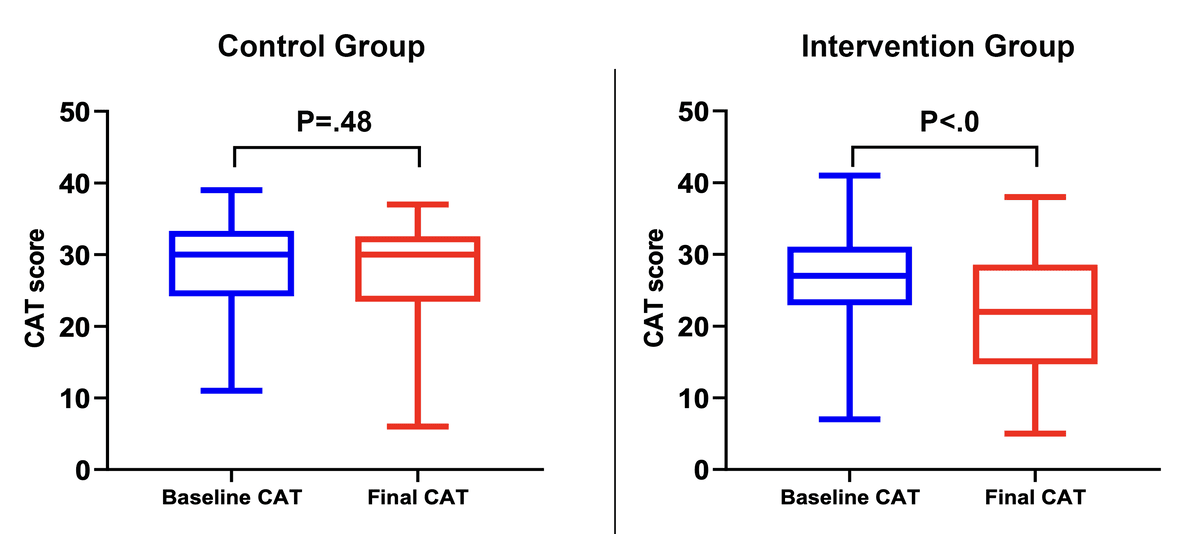
Comparison of chronic obstructive pulmonary disease assessment test scores at baseline and at the end of follow-up for control and intervention group patients included in the study. CAT: chronic obstructive pulmonary disease assessment test.

Regarding the impact of COPD on quality of life measured with the CAT questionnaire, although no differences were found in the score between the baseline measurement and at the end of follow-up in the control group, a significant decrease was observed in the intervention group (median baseline CAT 27, IQR 23-31 vs median final CAT 22, IQR 14-28; *P*<.001; Figure 4). In addition, the CAT score was significantly higher in patients undergoing conventional follow-up compared with those followed with the mobile app (median 30, IQR 23-32 vs median 22 IQR 14-28; *P*=.02).

When evaluating the record of HO therapy prescriptions in each group, it was found that the proportion of individuals who performed the HO therapy prescriptions correctly was higher in the IG throughout each month of follow-up. The frequency of accessing tutorials or educational records was significantly higher in the IG (95.7% vs 59%; *P*=.003) in the first month of follow-up (Table 4). Furthermore, during the study period, 50% (n=11) of participants in the IG used the mobile app for more than 21 days (median 21, IQR 16-28). The median was greater than 14 days for most variables requiring frequent recording or entry: vital signs recording (median 19, IQR 15-27), Borg scale recording (median 16, IQR 14-22), and oxygen prescription recording (median 14, IQR 11-18; Multimedia Appendix 4).

Satisfaction was mostly observed among users of the AppO2 app, including patients, caregivers, and therapists. Details regarding the measurement of satisfaction with the AppO2 app are presented in Multimedia Appendix 5.

Similarly, in interviews assessing the perception and acceptance of technology using the TAM, the IG reported that the use of the mobile app fostered confidence and improved communication with health care professionals. The group emphasized the value of notifications regarding their vital signs, noting that having access to this information made them more responsible in managing and caring for their disease. Multimedia Appendix 6 presents some expressions reflecting the perceptions and acceptance of AppO2.

**Table 4:** Comparison of oxygen therapy recording, education, and oxygen cylinder duration in the control group versus the intervention group.

Variable				Intervention (n=23), n (%)		Control (n=22), n (%)		*P* value
**Records oxygen prescription**								
		Month 1	22 (96)		14 (64)		.006	
		Month 2	22 (96)		11 (50)		.002	
		Month 3	22 (96)		10 (48)		.001	
**Records education or reviews tutorials**								
		Month 1	22 (96)		13 (59)		.003	
		Month 2	14 (61)		9 (41)		.18	
		Month 3	4 (17)		14 (67)		.002	
**Records duration of oxygen cylinder**								
	Month 1			6 (26)		2 (9)		.13
	Month 2			4 (17)		3 (14)		.72
	Month 3			5 (17)		7 (33.)		.44

Finally, the binary logistic regression model was statistically significant (chi-square value=5.9, *P*<.001) and demonstrated a good fit (Hosmer-Lemeshow test=3.92, *P*=.80). The use of the mobile app was the only variable independently associated with the impact on the quality of life of patients with COPD, as measured using the CAT questionnaire (adjusted OR 0.15; 95% CI 0.02-0.82; Table 5).

**Table 5:** Use of AppO2 and its association with decreased impact of chronic obstructive pulmonary disease on quality of life measured by chronic obstructive pulmonary disease assessment test.

Variable		Crude OR^a^ (95% CI)	*P* value	Adjusted OR (95% CI)	*P* value
**App usage**					
	Yes	0.15 (0.02-0.80)	.02	0.15 (0.02-0.82)	.02
	No	6.57 (1.21-35.5)	.02	6.57 (1.21-35.52)	.02
**Sex**					
	Male	0.79 (0.18-3.30)	.75	1.09 (0.18-6.38)	.92
	Female	1.25 (0.29-5.20)	.75	0.91 (0.15-5.34)	.92
**Oxygen use duration**					
	More than 15 hours	0.73 (0.17-3.00)	.66	0.58 (0.10-3.28)	.53
	Less than 15 hours	1.36 (0.33-5.50)	.66	1.72 (0.30-9.70)	.53
**Smoking duration**					
	More than 15 years	1.6 (0.35-7.20)	.54	—^b^	—
	Less than 15 years	0.62 (0.13-2.82)	.54	—	—
**Smoking and biomass exposure**					
	Yes	2.1 (0.51-8.70)	.29	—	—
	No	0.46 (0.11-1.90)	.29	—	—

^a^OR: odds ratio.

^b^Not applicable.

## Discussion

### Overview

A 2-arm randomized clinical trial was conducted to determine the effectiveness and acceptability of the AppO2 mobile app for managing HO therapy in patients with COPD in Cali, Colombia.

The use of health technology tools that offer clinical interventions is emerging as a promising strategy in the health care setting [38,39]. In recent years, self-management interventions supported by smartphone apps have been shown to improve disease management in chronic patients, thus decreasing hospitalizations and emergency room visits and improving quality of life [40].

### Quality of Life of Patients With COPD Receiving Home Oxygen When Using AppO2

The main finding of this investigation is that the use of the AppO2 app is associated with a decrease in the score of the impact of COPD on quality of life. This reduction in the CAT score suggests a lower negative impact of the disease on the quality of life of patients and an improvement in their general well-being. We believe that this finding is related to the user-centered design approach of AppO2 [19], which addressed specific needs expressed by patients and their caregivers during the initial stages of the project. Therefore, this app is not only limited to being a clinical monitoring tool, but it also promotes the development of self-care skills and recognition of clinical signs. Wang et al [41], who also used the CAT to assess the impact on patient’s quality of life, observed significant improvements in patients who used a mobile app compared with those who received conventional medical care for COPD.

Another important aspect is the improvement in the dyspnea score according to the Borg scale observed in the IG. Participants mentioned that the information provided by AppO2 through images and notifications when determining the degree of dyspnea allowed them to establish a direct relationship with their perception of effort and respiratory difficulty. They also stated that this facilitated communication with therapists through the mobile app and during home visits. These findings are consistent with those reported by Kayyali et al [42], who demonstrated that patients who received clinical information through technological tools, such as mobile apps, could establish correlations with their symptoms and determine when to contact health care professionals.

A Cochrane review [43] suggested that digital tools in health care might improve patients’ quality of life, although long-term effects remain unclear. Prolonged use of these digital interventions could improve symptoms such as dyspnea. Our findings align with studies [44,45] that indicate mHealth apps enhance patients’ quality of life.

#### Health Perception and Self-Care of Patients With COPD Receiving Home Oxygen When Interacting With AppO2

Our study found no significant differences between the groups regarding the recording of the clinical signs such as oxygen saturation, respiratory rate, and heart rate. This might be due to the chronic condition [38,40] of the patients, which did not show notable clinical changes during follow-up. However, users in the IG reported that AppO2 improved their health and confidence in recognizing and interpreting vital signs. This suggests that the usefulness of the mobile app extends beyond clinical sign monitoring, positively impacting patients’ self-care and their perception of the app’s use, as supported by other research on mHealth technologies [42,46,47].

Another point worth mentioning is the interaction of the patients with the oxygen prescription section. The few patients in the IG functionality found it helpful for managing their treatment autonomously by viewing their oxygen prescription at any time, facilitating the use of the HO. Despite previous positive feedback on this feature [4,19], its low usage in this study might warrant its reconsideration or removal. Conversely, the CG only had access to this information during home visits from health care professionals.

Furthermore, patients who used AppO2 mentioned feeling more responsible and confident in managing their disease, emphasizing that access to clinical information aided them in adhering to guidelines for monitoring their clinical signs. This finding aligns with previous investigations [43,47], which indicate that patients with chronic diseases consider the use of digital tools for self-care as a good strategy to access clinical information and optimize disease management.

#### Acceptability and Connection Time With AppO2

According to the results of the level of acceptability measured using the TAM tool, most patients expressed being completely satisfied with the usefulness and ease of use of AppO2. In addition, patients in the IG expressed feeling safe when using the app. These results are consistent with those of the investigation conducted by Knox et al [16], who designed an app for patients with COPD also using the TAM. They observed that patients demonstrated high motivation and a positive attitude toward using the app, especially in making health-related decisions [16].

On the other hand, while the average use of the app for 21 days over a 3-month period may seem small, it is important to consider that its design does not require continuous daily use to be effective. The app focuses on building patient confidence and promoting self-awareness of their symptoms. Through intermittent but strategic use, patients acquire key tools to monitor and manage their condition more autonomously, potentially contributing to a better quality of life. On the other hand, patients mentioned in interviews that they felt more secure and confident knowing that therapists could access their records at any time, assess their clinical condition, and adjust treatment if necessary. This sense of constant accompaniment helped them to feel “less alone” in the process of their treatment, enhancing their mental well-being.

Likewise, some authors have indicated that certain factors may influence the frequency of AppO2 use. These factors highlight the acquisition of skills and knowledge for self-reporting and managing their disease, which decreases the need to consult tutorials or informative sections. As patients become more familiar with these skills, they experience greater autonomy, leading to more selective use of the app’s functions, focusing on those they consider most relevant to their daily routine [48,49].

In this sense, the trend of AppO2 use is reflected in the maximum period of disconnection of 2 weeks observed among the participants, demonstrating a significant engagement with the app. Patients expressed greater confidence and education in recognizing their vital signs. Thus, effective use of AppO2 is not only measured by frequency of access but also by its ability to foster self-management and clinical monitoring.

In previous studies [50-52], it has been documented that adherence to mHealth apps tends to decline over time, especially after an initial phase of intensive use. Perski et al [51] highlight that this decline in user engagement may influence clinical outcomes. However, they also identify factors that may prolong use, such as personalization and regular reminders. Similarly, Byambasuren et al [52] noted that the “novelty” of mobile apps often wears off after the first few months. However, they suggest that intuitive design [53] and ongoing support from health professionals may improve long-term user retention.

In our study, the median number of app logins was 21 days during the 3-month follow-up period. In addition, we observed that the maximum time offline was 10 days. These data reflect a pattern of use that aligns with the app’s design and does not require continuous daily access to be effective. The design’s simplicity [53], vital sign notifications, and ease of use have been key factors in maintaining patient engagement.

As mentioned earlier, access to the tutorials in the IG decreased by the third month. The authors attribute this finding to the fact that the content of the videos did not vary over time, which may have led patients to view the education provided as adequate and integrated into their activities. This allowed them to be more selective in choosing the sections that best suited their needs and daily routine. In this sense, it is crucial to explore reinforcement strategies in future studies to ensure continued app use and maximize its clinical impact. This emphasis on continuous improvement in app usage is essential for the long-term success of such interventions [54,55].

Finally, the IG showed a maximum disconnection period of 2 weeks, indicating that the patients continuously interacted with the app. This adherence may be attributed to the high usability of AppO2, achieved through the end-user-centered design methodology applied in this research [4,18,19]. Conversely, the viewing of tutorials decreased in the final month of follow-up in the IG, which could be attributed to patients becoming more adept at self-monitoring and recognizing their vital signs. Similarly, Ding et al [56] reported that the use of the mobile app was more frequent during the first week of launch but decreased over time. Similarly, previous research [48,49] observed that users stop consulting some functions of mobile apps over time. Some factors that may influence this are the acquisition of skills and knowledge for self-registration and management of their disease, which reduces the need to consult tutorials or information sections. The continuous use of the app can also lead to a more selective use of its functions, focusing on those that are essential in their daily routine [48,49].

### Principal Results

The findings of this study indicate that the use of the “AppO2” mobile app is associated with a significant improvement in the quality of life for patients with COPD and HO therapy. The high acceptability of the app is evidenced by a notable degree of satisfaction and adherence among users. Furthermore, “AppO2” not only facilitates self-care by enabling patients to manage their treatment more autonomously but also enhances their confidence in self-managing their condition. This contributes to better adaptation to treatment and greater self-care capabilities.

### Limitations

The sample size comprised 45 participants. To avoid a lack of statistical power, the study included 3 home care institutions, which helped increase participant representativeness and the generalizability of the results for home health programs.

A limitation of the study is the 3-month duration. Although this period was sufficient to record favorable changes in the CAT score, which measures the impact of COPD on patients’ quality of life, as well as their self-care and health perceptions, a longer follow-up could provide insights into the long-term effects.

The ANCOVA results indicated that the use of the app reduced dyspnea, as measured by the Borg scale, at month 3. However, the statistical significance of the initial Borg score (Borg month 1) suggests that the initial levels of dyspnea influenced the final results. The influence of the covariate suggests that other unmeasured variables, such as disease severity or comorbidities, may have affected the results, although this factor was controlled for in the analysis. Similarly, another possible limitation is the Hawthorne effect or observation bias, which occurs when study participants can systematically alter their behavior when they know they are being observed or monitored [50]. This, along with the inability to blind participants, may have influenced participant behavior. Future research should consider more discreet observation methods to minimize these biases.

### Conclusions

The use of AppO2 is associated with a better quality of life in patients with COPD receiving HO therapy. The acceptability results for AppO2 show a high degree of satisfaction and adherence to its use. In addition, this mobile app promotes self-care and allows patients to develop confidence in managing their disease.

## Appendix

Multimedia Appendix 1 Adaptation of the AppO2 acceptance questionnaire (dimension and item).

Multimedia Appendix 2 Questions on the perception of the AppO2 mobile app.

Multimedia Appendix 3 CONSORT-eHEALTH checklist (V 1.6.1).

Multimedia Appendix 4 Use of the mobile app by patients in the intervention group (n=23).

Multimedia Appendix 5 Satisfaction of patients, caregivers, and therapists using the mobile app for home oxygen therapy monitoring (n=24). (A) Perceived usefulness. (B) Perceived ease of use. Question 1: Using this app helps me complete my tasks more quickly. Question 2: Using this app enhances my performance. Question 3: Using this app increases my productivity. Question 4: Using this app enhances the effectiveness of my work or self-care. Question 5: Using this app makes it easier for me to perform my work or self-care. Question 6: I find this app very useful for my work or self-care. Question 7: Learning to use this app was easy for me. Question 8: I find this app easy to do what I need to do. Question 9: My interaction with this app was clear and understandable. Question 10: I find this app flexible to interact with. Question 11: It will be easy for me to become an expert in using the app. Question 12: I find this app easy to use.

Multimedia Appendix 6 Technology Acceptance Model and AppO2 perception interviews.

## Abbreviations

ANCOVA: analysis of covariance
CAT: chronic obstructive pulmonary disease assessment test
CG: control group
COPD: chronic obstructive pulmonary disease
HO: home oxygen
IG: intervention group
mHealth: mobile health
OR: odds ratio
TAM: technology acceptance model
