# Supplementary material for: Efficacy and Acceptability of a Mobile App for Monitoring the Clinical Status of Patients With Chronic Obstructive Pulmonary Disease Receiving Home Oxygen Therapy: Randomized Controlled Trial
Source: J Med Internet Res. 2025 Jan 6;27:e65888. doi: 10.2196/65888 (PMC11747540; doi:10.2196/65888)
Supplement: Multimedia Appendix 1 [file jmir_v27i1e65888_app1.pdf]

Multimedia Appendix 1. Adaptation of the AppO2 acceptance questionnaire (dimension and item).

Perception of usefulness

- Using this app helps me perform my activities faster
- Using this app improves my performance
- Using this app increases my productivity
- Using this app enhances the effectiveness of my work or self-care
- Using this app makes it easier for me to complete my work or self-care
- I find this app very useful for my work or self-care

Perception of ease

- I find this app easy to use for completing my tasks
- My interaction with this app was clear and understandable
- I find this app flexible for interacting with it
- It will be easy for me to become an expert in using the app
- Learning to use the app was easy for me
- I find this app easy to use
