# Supplementary material for: Efficacy and Acceptability of a Mobile App for Monitoring the Clinical Status of Patients With Chronic Obstructive Pulmonary Disease Receiving Home Oxygen Therapy: Randomized Controlled Trial
Source: J Med Internet Res. 2025 Jan 6;27:e65888. doi: 10.2196/65888 (PMC11747540; doi:10.2196/65888)
Supplement: Multimedia Appendix 2 [file jmir_v27i1e65888_app2.pdf]

Multimedia Appendix 2. Questions on the perception of the AppO2 mobile app.

Questions for patients

- What did you like most about AppO2? Why?
- What did you like least about AppO2? Why?
- Which section or feature of AppO2 did you like the most? Why?
- Which activity in AppO2 have you benefited from the most? Why?
- Which activity in AppO2 have you benefited from the most? Why?
- Do you feel secure using AppO2? Why?
- Has the use of AppO2 facilitated home oxygen management? Why?
- Has AppO2 been supportive in the treatment and medical prescription of home oxygen? Why?

Questions for professionals

- What did you like most about AppO2? Why?
- What did you like least about AppO2? Why?
- Which section or feature of AppO2 did you like the most? Why?
- Has the use of AppO2 allowed you to make timely decisions regarding home oxygen management? Why?
- Which activity in AppO2 have you benefited from the most? Why?
- Do you feel secure using AppO2? Why?
- Has the information provided by AppO2 been useful? Why?
- Has it been easy to interpret clinical data through AppO2?
