# Supplementary material for: Efficacy and Acceptability of a Mobile App for Monitoring the Clinical Status of Patients With Chronic Obstructive Pulmonary Disease Receiving Home Oxygen Therapy: Randomized Controlled Trial
Source: J Med Internet Res. 2025 Jan 6;27:e65888. doi: 10.2196/65888 (PMC11747540; doi:10.2196/65888)
Supplement: Multimedia Appendix 4 [file jmir_v27i1e65888_app4.pdf]

Multimedia Appendix 4. Use of the mobile app by patients in the intervention group (n=23).

Variable	Mean (IQR[a])
Number of days using the app	21 (16-28)
Number of vital signs entries	19 (15-27)
Number of Borg Scale entries	16 (14-22)
Number of tutorial entries	12 (7-15)
Number of dose counter entries	10 (6-15)
Number of oxygen prescription entries	14 (11-18)
Maximum time offline	10 (9-14)
