# Supplementary figures and images for: Efficacy and Acceptability of a Mobile App for Monitoring the Clinical Status of Patients With Chronic Obstructive Pulmonary Disease Receiving Home Oxygen Therapy: Randomized Controlled Trial
Source: J Med Internet Res. 2025 Jan 6;27:e65888. doi: 10.2196/65888 (PMC11747540; doi:10.2196/65888)

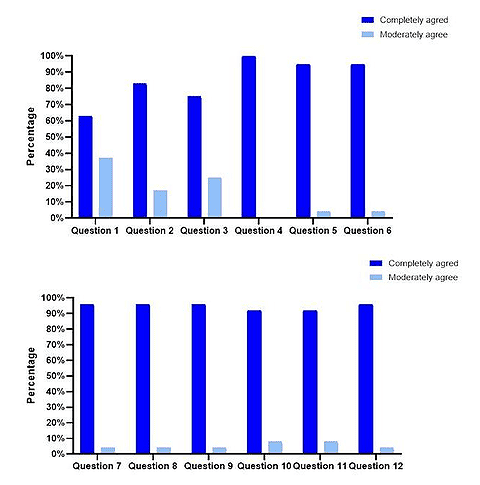

Supplement: Multimedia Appendix 5 [file jmir_v27i1e65888_app5.png]
