# Supplementary material for: Efficacy and Acceptability of a Mobile App for Monitoring the Clinical Status of Patients With Chronic Obstructive Pulmonary Disease Receiving Home Oxygen Therapy: Randomized Controlled Trial
Source: J Med Internet Res. 2025 Jan 6;27:e65888. doi: 10.2196/65888 (PMC11747540; doi:10.2196/65888)
Supplement: Multimedia Appendix 6 [file jmir_v27i1e65888_app6.pdf]

Multimedia Appendix 6. Technology Acceptance Model and AppO2 perception interviews.

Participants	Verbatim statements of participants	Dimension
Patients [P]	• *I feel that what I liked most about the application… was that I could know how my father was, his vital signs, and… how to learn how I can measure those signs. I found it very novel, safe, easy… and useful to know how the dyspnea is… and to know how to give him oxygen until the therapist comes to the visit.* [P20]	Ease of use
	• *I really liked this application… Above all, the notifications of the vital signs… and being able to see what the therapist prescribed for oxygen, if… I forgot what the therapist said, I could see it in the app. I found it easy to use. I would like to use it without the internet… Also, I feel that with this app, my health is better.* [P12]	Usefulness
	• *First, I found this app to be safe and easy to use… because while the health care professional arrives, I can see how my mother is… I also liked the images and videos —they are very clear to understand.* [P5]	Ease of use
	• *The part that I used the most… dyspnea and vital signs, seemed very useful to us… Also, the videos were very easy to understand. I really liked it; it was very innovative, and I would like to continue using it. I feel that, in general, I am stable...* [P17]<br>• *… what I like the most is the information in the videos. I feel that I have learned things that can help me feel better… and also to tell the therapist how I feel.* [8]	Usefulness
Health care professional [HCP]	• *Yes, the app seemed very good to me—safe and easy to keep records… what I liked the most was the oxygen prescription section, so I could have quick access to the signs and make decisions regarding management or behavior with the patients.* [HCP1]	Usefulness and ease of use
	• *This application allowed me to have better communication with the patients… even when I did not go to their home, I could review the records and view the clinical progress. The follow-up with this app was easy and safe… it allowed me to make decisions based on the records with quick access to the information.* [HCP2]	Utility and ease of use
